# Indirect Associations Between Perceived Coach–Athlete Relationship Quality and Mental Health Through Competitive State Self-Confidence: A Cross-Sectional Study Among Hungarian Athletes

**DOI:** 10.3390/sports14090416

**Published:** 2026-09-21

**Authors:** Nóra Papp, Tamás Berki, Renátó Tóth, Rita Géczi, László Tóth

**Affiliations:** 1 School of Doctoral Studies, Hungarian University of Sport Science, 1123 Budapest, Hungary; papp.nora@tf.hu (N.P.); geczi.rita@tf.hu (R.G.); 2 Department of Physical Education Theory and Methodology, Hungarian University of Sport Science, 1123 Budapest, Hungary; 3 Independent Researcher, 1123 Budapest, Hungary; tothrenato29@gmail.com; 4 Department of Sport Games, Hungarian University of Sports Science, 1123 Budapest, Hungary; 5 Department of Psychology and Sport Psychology, Hungarian University of Sports Science, 1123 Budapest, Hungary; toth.laszlo@tf.hu; 6 Teacher Training Institute, Hungarian University of Sport Science, 1123 Budapest, Hungary; 7 Institute of Health Promotion and Sport Sciences, Faculty of Education and Psychology, Eötvös Loránd University, 1117 Budapest, Hungary

**Keywords:** coach–athlete relationship, perceived coach–athlete relationship quality, competitive state self-confidence, positive mental health, athlete well-being, mediation analysis, sport psychology

## Abstract

Mental health in athletes is shaped by both interpersonal and intrapersonal psychological resources. This study examined whether the association between athletes’ perceptions of the coach–athlete relationship and emotional, psychological, and social well-being showed statistically significant indirect associations through competitive state self-confidence. A cross-sectional sample of 211 adult Hungarian athletes (149 women and 62 men; mean age = 23.39 years, SD = 3.58) completed validated Hungarian measures of the coach–athlete relationship, competitive state self-confidence, and positive mental health. Participants were recruited online by distributing the questionnaire directly to athletes and through sports clubs. Three separate single-mediator models were estimated. Athletes’ perceptions of the coach–athlete relationship were positively associated with competitive state self-confidence (β = 0.41, *p* < 0.001), which was positively associated with emotional (β = 0.26, *p* < 0.001), psychological (β = 0.30, *p* < 0.001), and social well-being (β = 0.19, *p* = 0.010). Significant indirect associations through competitive state self-confidence were observed for emotional (β = 0.11, *p* = 0.002), psychological (β = 0.12, *p* < 0.001), and social well-being (β = 0.08, *p* = 0.016), whereas the corresponding direct associations were not statistically significant. These findings indicate that the observed associations were statistically consistent with indirect associations through competitive state self-confidence. However, the cross-sectional design does not establish temporal ordering or a causal mediating mechanism. Longitudinal and intervention-based research is needed to determine the temporal direction of these associations.

## 1. Introduction

Mental health is a fundamental component of athletic functioning and long-term sporting success, yet it has historically received less systematic attention than physical health and performance in sport science [1]. Athletes may experience performance pressure, injury, selection uncertainty, organizational demands, career transitions, and difficulties balancing sporting and non-sporting responsibilities [1,2]. These demands may increase athletes’ vulnerability to mental health symptoms and disorders, particularly when adequate personal, interpersonal, and organizational resources are unavailable [1,2]. At the same time, supportive interpersonal relationships have been positively associated with athletes’ well-being and negatively associated with anxiety, depression, and perceived stress [3]. Accordingly, understanding the psychological and interpersonal factors that may protect and promote athletes’ mental health has become a central concern for sport science and applied practice [1,2].

Beyond the presence or absence of clinical symptoms, researchers and practitioners have increasingly focused on the mental performance competencies that allow athletes to function effectively under the demands of competitive sport. This shift is reflected in the Gold Medal Profile for Sport Psychology (GMP-SP), a comprehensive, evidence-informed framework developed in collaboration with Canadian high-performance sport organizations [4].

The GMP-SP conceptualizes fundamental, self-regulatory, and interpersonal competencies as interconnected resources operating within the athlete’s performance environment [4]. Of particular relevance to the present study, the framework proposes that interpersonal competencies, including the coach–athlete relationship, are related to fundamental psychological competencies such as confidence [4]. However, the statistical relationships among coach–athlete relationship quality, competitive state self-confidence, and positive mental health have not yet been sufficiently examined within an integrated model.

Among the fundamental competencies included in the GMP-SP, confidence occupies a particularly prominent position [4]. In the present study, confidence was operationalized specifically as competitive state self-confidence, referring to athletes’ positive expectations and perceived ability to succeed in an upcoming competitive situation [5,6].

For conceptual clarity, general sport confidence is understood here as a relatively enduring belief in one’s ability to succeed across different sporting situations, whereas self-efficacy refers more narrowly to confidence in one’s ability to perform a particular task or action. Competitive state self-confidence differs from both constructs because it reflects athletes’ confidence regarding an imminent competitive situation. Thus, the variable examined in the present study is situation-specific, whereas emotional, psychological, and social well-being represent broader aspects of psychological functioning.

An additional conceptual consideration concerns the different temporal reference periods of the measures. Competitive state self-confidence refers to an upcoming competitive situation, whereas the MHC-SF assesses well-being experienced during the previous month. This temporal mismatch may limit the conceptual alignment of the variables; therefore, the estimated indirect associations should be interpreted as cross-sectional statistical relationships between constructs measured over different time frames rather than as evidence that competitive state self-confidence temporally mediates broader well-being.

Competitive state self-confidence may be influenced by athletes’ performance accomplishments, physical and mental preparation, social support, and coaches’ leadership and behavior [7]. Within this framework, athletes’ perceived training status may represent an important source of competition-specific confidence. Favorable results in fitness, technical, or sport-specific performance assessments may provide concrete evidence of readiness and strengthen athletes’ beliefs that they are capable of meeting competitive demands. Coaches may reinforce these appraisals by helping athletes interpret assessment results, recognize improvements, and establish realistic expectations for target competitions. This process may be particularly relevant in professional and high-performance sport, where preparation is frequently organized around specific target events and athletes are required to perform under substantial pressure. Nevertheless, training status and performance-assessment results were not measured in the present study; therefore, this interpretation was not tested empirically.

More recent meta-analytic evidence has confirmed a positive association between athletes’ self-confidence and sport performance, although the strength of this relationship may vary according to the measurement approach, sport context, and level of competition [8]. Importantly, competitive state self-confidence is not exclusively an intrapersonal resource because athletes’ beliefs about their ability to succeed in an upcoming competition may also be shaped by the feedback, support, recognition, and leadership they receive from their coaches. Recent evidence indicates that perceived coach support is positively associated with the quality of the coach–athlete relationship, athletes’ self-confidence, and psychological well-being [9].

Coaching quality is a broader, multidimensional concept that can involve technical and pedagogical expertise, planning, leadership, feedback behaviors, the motivational climate, and characteristics of the athlete and sport context. The coach–athlete relationship represents one interpersonal component of this broader concept rather than a comprehensive or objective indicator of overall coaching effectiveness. The present study therefore focuses specifically on athletes’ perceptions of their relationship with the coach, as these relational experiences may be particularly relevant to athletes’ confidence and well-being.

Coach–athlete relationship quality is commonly conceptualized through three interrelated dimensions: closeness, commitment, and complementarity. Closeness reflects the affective bond between the coach and athlete, commitment refers to their intention to maintain the relationship, and complementarity describes their cooperative and responsive interactions. Together, these dimensions characterize the affective, cognitive, and behavioral aspects of athletes’ perceived relationships with their coaches [10]. Recent studies further indicate that the quality of the coach–athlete relationship and the support provided by coaches are associated with athletes’ self-confidence and psychological well-being [9,11].

Competitive state self-confidence was selected as the intervening variable because it represents a situation-specific athlete appraisal that may be closely associated with the feedback, support, and recognition received from the coach, while also being relevant to broader psychological functioning. Theoretically, confidence regarding an imminent competition may be associated with emotional well-being through more positive affect and reduced uncertainty, with psychological well-being through perceived competence and purpose, and with social well-being through confidence in one’s role and interactions within the sporting environment. The proposed model therefore examines whether the associations between perceived coach–athlete relationship quality and the three dimensions of well-being are statistically consistent with indirect associations through competitive state self-confidence rather than being limited to separate bivariate correlations. However, it remains unclear whether competitive state self-confidence statistically accounts for part of the association between athletes’ perceptions of the coach–athlete relationship and positive mental health.

Building on the GMP-SP proposition that interpersonal competencies are related to fundamental competencies relevant to athletes’ mental health [4], the present study examined whether statistically significant indirect associations between athletes’ perceptions of coach–athlete relationship quality and emotional, psychological, and social well-being were observed through competitive state self-confidence in a sample of Hungarian athletes. Mental health was conceptualized according to Keyes’s multidimensional model and operationalized in terms of emotional, psychological, and social well-being [12].

Three hypotheses were formulated: (H1) athletes’ perceptions of coach–athlete relationship quality would be positively associated with competitive state self-confidence; (H2) competitive state self-confidence would be positively associated with emotional, psychological, and social well-being; and (H3) statistically significant indirect associations between perceived coach–athlete relationship quality and each dimension of well-being would be observed through competitive state self-confidence. Given the cross-sectional design, H3 concerns statistically significant indirect associations and does not imply temporal mediation or establish a causal sequence among the variables.

## 2. Materials and Methods

### 2.1. Participants

The sample consisted of N = 211 Hungarian athletes (149 female, 62 male), with a mean age of 23.39 years (SD = 3.58). To be eligible, participants had to be at least 18 years old, be formally registered in a sport, and report that they were currently participating in sport. Respondents who indicated that they were not currently participating in sport were excluded. No minimum requirement was specified regarding weekly training volume or years of training or competitive experience, and having a current coach was not a formal eligibility criterion. An a priori power analysis was not conducted. The sample size was determined by the number of eligible athletes who provided complete responses during the data-collection period, reflecting a convenience-sampling approach. The resulting sample of 211 participants was considered adequate for estimating the prespecified single-mediator models and their indirect effects with 95% confidence intervals. Nevertheless, the absence of an a priori sample-size calculation is acknowledged as a methodological limitation.

Participants represented both team (n = 101) and individual (n = 110) sports. Athletes competing in team sports had a mean age of 23.01 years (SD = 3.25), whereas those in individual sports had a mean age of 23.78 years (SD = 3.82). On average, participants reported 11.75 h of training per week (SD = 8.94). Competitive level was self-reported using predefined response categories. Professional athletes reported that sport was their occupation and a source of income (n = 35); semi-professional athletes reported earning income from sport while also working in another occupation (n = 92); and amateur athletes reported receiving no income from sport and/or participating at a recreational level (n = 84). The demographic and sport-related characteristics ofb the participants are summarized in Table 1.

No operational definitions were provided for these categories; therefore, they reflect participants’ self-classification and were used only for descriptive purposes. The most frequently represented sports were tennis (n = 46) and football (n = 43).

### 2.2. Procedure

Data were collected using an online questionnaire battery administered via Google Forms (Google LLC, Mountain View, CA, USA). Participant recruitment and data collection took place between October 2024 and October 2025. The questionnaire battery was designed to assess athletes’ motivation, well-being, confidence, resilience, and perceptions of the coach–athlete relationship within a cross-sectional, self-report survey design. Participants were recruited using a non-probability convenience-sampling approach. The online questionnaire link was distributed directly to athletes and to sports clubs, which were asked to circulate the survey among potentially eligible athletes. Before participation, all participants received information about the purpose and procedures of the study and provided electronic informed consent. Participation was voluntary and anonymous, and respondents completed the survey at their convenience. Completion of the questionnaire battery was estimated to require approximately 20–25 min. All scale items and variables included in the present analyses were set as required in Google Forms. One ancillary background item concerning concussion history was optional and was not included in the analyses. Accordingly, no missing data were present for the analytic variables, and no missing-data treatment or imputation was required.

Eligibility was assessed using participants’ self-reported age, formal registration in a sport, and current sport participation. All 211 submitted questionnaires met the eligibility criteria, and no responses were excluded. Because the survey was anonymous and Google Forms was not configured to limit submission to one response per person, potential duplicate submissions could not be verified using personal identifiers, and no formal duplicate-response screening was conducted. Individual completion duration could not be objectively evaluated because survey start times were not recorded. Google Forms recorded only submitted questionnaires; therefore, the number of individuals who accessed or started the survey without submitting a response was not available. Consequently, the final analytic sample consisted of 211 participants. The study was conducted in accordance with the Declaration of Helsinki and approved by the Ethics Committee of the Hungarian University of Sports Science (Approval No. MTSE-OKE-KEB/03/2023). All responses were handled confidentially in accordance with applicable ethical and data-protection requirements.

### 2.3. Measures

#### 2.3.1. Perceived Coach–Athlete Relationship Quality

Perceived coach–athlete relationship quality was assessed using the validated Hungarian athlete version of the 11-item Coach–Athlete Relationship Questionnaire [10,13]. The CART-Q assesses athletes’ direct perceptions of the quality of their relationship with their coach across three dimensions: closeness, reflecting an affective bond characterized by trust, respect, and appreciation; commitment, referring to the intention to maintain the coach–athlete relationship over time; and complementarity, representing cooperative, responsive, and coordinated coach–athlete interactions. The questionnaire comprises four items assessing closeness, three items assessing commitment, and four items assessing complementarity. Items are rated on a seven-point Likert-type scale ranging from 1 (“strongly disagree”) to 7 (“strongly agree”), with higher scores indicating a more positive perception of the coach–athlete relationship. Previous psychometric evaluations have supported the factorial validity, internal consistency, and cross-cultural applicability of the 11-item athlete version of the CART-Q [10,14]. The Hungarian adaptation also demonstrated adequate factorial validity, internal consistency, and convergent validity among Hungarian athletes [13]. Following previous research in which the three relationship dimensions were operationalized as an overall indicator of relationship quality [15], the items within each CART-Q dimension were first averaged to calculate separate closeness, commitment, and complementarity scores. The mean scores for the three dimensions were then summed to create a composite perceived coach–athlete relationship quality score. The resulting composite score had a theoretical range from 3 to 21, with higher scores indicating a more positive athlete-perceived coach–athlete relationship. In the present sample, the composite perceived coach–athlete relationship quality score demonstrated excellent internal consistency (α = 0.97).

#### 2.3.2. Competitive State Self-Confidence

Competitive state self-confidence was assessed using the six-item self-confidence subscale of the revised Hungarian version of the Competitive State Anxiety Inventory-2 (CSAI-2) [5,6]. The subscale assesses athletes’ positive expectations and perceived ability to succeed in a competitive situation. Participants were instructed to indicate how they felt at the time of questionnaire completion. If they were not immediately before a competition or match, they were instructed to consider how they generally felt before competition. Because participants completed the questionnaire at a time of their choice, temporal proximity to a specific competition was not recorded or standardized. Items are rated on a four-point Likert-type scale ranging from 1 (“not at all”) to 4 (“very much so”), with higher scores indicating greater competitive state self-confidence. The six self-confidence items (items 3,6,9,12,15, and 18) were averaged, resulting in a theoretical score range from 1 to 4. The revised Hungarian version demonstrated an adequate factorial structure, internal consistency, and test–retest reliability among Hungarian athletes [6]. In the present sample, the competitive state self-confidence subscale demonstrated good internal consistency (α = 0.85).

#### 2.3.3. Mental Health (Emotional, Psychological, and Social Well-Being)

Athletes’ positive mental health was assessed using the validated Hungarian version of the 14-item Mental Health Continuum–Short Form [16,17]. The MHC-SF is based on Keyes’s multidimensional model of positive mental health and measures three dimensions of well-being: emotional well-being, psychological well-being, and social well-being [12]. The scale comprises three items assessing emotional well-being, six items assessing psychological well-being, and five items assessing social well-being. Participants indicated how frequently they had experienced each aspect of well-being during the previous month using a six-point Likert-type scale ranging from 1 (“never”) to 6 (“every day”), with higher scores indicating higher levels of well-being.

Items within each dimension were summed to calculate separate emotional, psychological, and social well-being scores. The score varied between 0–6. The Hungarian version demonstrated adequate factorial validity, internal consistency, and convergent and discriminant validity in a Hungarian adult sample [17]. In the present sample, internal consistency was good for emotional well-being (α = 0.87) and psychological well-being (α = 0.83), and acceptable for social well-being (α = 0.75).

### 2.4. Statistical Analysis

Descriptive statistics were calculated for the main study variables, including means and standard deviations. No missing data were present in the analytic dataset; therefore, no missing-data treatment or imputation was required. Internal consistency was assessed using Cronbach’s alpha. Pearson correlations were computed to examine associations among the variables. To test the hypothesized mediation model, three separate single-mediator analyses were performed. The three single-mediator models were estimated in jamovi 2.6 (The jamovi project, Sydney, Australia) using the GLM Mediation Model procedure in the jAMM (Advanced Mediation Models) module (Marcello Gallucci, University of Milano-Bicocca, Milan, Italy). Model parameters were estimated using maximum likelihood estimation. Because the sampling distribution of an indirect effect may be non-normal, 95% confidence intervals for the indirect effects were calculated from 5000 nonparametric bootstrap resamples using the percentile method. An indirect association was considered statistically supported when its bootstrap confidence interval did not include zero. In each model, perceived coach–athlete relationship quality was entered as the independent variable, competitive state self-confidence as the mediator, and one dimension of mental health—emotional, psychological, or social well-being—was entered as the outcome variable. Indirect, direct, and total effects were estimated for each model. Unstandardized effect estimates (B) with 95% confidence intervals and standardized coefficients (β) were reported. The direction, magnitude, and precision of the estimated effects were treated as the principal inferential information, with *p*-values reported as supplementary evidence. Prior to the inferential analyses, the assumptions underlying the Pearson correlations and the regression models used in the mediation analyses were evaluated. Scatterplots were inspected to assess the linearity of the associations between the continuous study variables and to identify potentially influential bivariate observations. For each regression equation constituting the mediation models, plots of standardized residuals against predicted values were examined to evaluate linearity and homoscedasticity. The distribution of residuals was assessed using histograms and normal Q–Q plots. Potentially influential observations were evaluated using standardized residuals, leverage values, and Cook’s distances. Multicollinearity was examined using tolerance and variance inflation factor (VIF) statistics in models containing both perceived coach–athlete relationship quality and competitive state self-confidence as predictors. The diagnostic procedures were conducted separately for the emotional, psychological, and social well-being models. No adjustment for multiple comparisons was applied because the three outcome-specific mediation models were specified a priori as components of H3 and represented conceptually distinct dimensions of the MHC-SF rather than exploratory tests of interchangeable outcomes. Nevertheless, the findings were interpreted while acknowledging the potential for an increased family-wise Type I error rate.

Statistical significance was set at *p* < 0.05. All statistical analyses were conducted using Jamovi 2.6 for macOS.

## 3. Results

### 3.1. Preliminary Analyses

The diagnostic analyses indicated that the assumptions underlying the Pearson correlations and regression-based mediation models were adequately satisfied. Visual inspection of the scatterplots supported approximately linear associations among the study variables and did not reveal clearly influential bivariate observations. Across the three outcome-specific models, inspection of the residual-versus-predicted-value plots did not indicate pronounced nonlinearity or heteroscedasticity, and the residual histograms and normal Q–Q plots indicated acceptable residual distributions. Examination of standardized residuals, leverage values, and Cook’s distances did not identify observations exerting undue influence on the model estimates. Multicollinearity was low in the regression equations containing both perceived coach–athlete relationship quality and competitive state self-confidence as predictors (VIF = 1.20; tolerance = 0.83). No observations were excluded on the basis of the diagnostic assessment.

Descriptive statistics, internal consistency coefficients, and zero-order correlations among the study variables are presented in Table 2. All measures demonstrated acceptable to excellent internal consistency. Perceived coach–athlete relationship quality was positively correlated with competitive state self-confidence and with the three dimensions of well-being. The corresponding correlation coefficient was numerically larger for competitive state self-confidence than for the well-being dimensions. Competitive state self-confidence was also positively correlated with emotional, social, and psychological well-being, with the numerically largest coefficient observed for psychological well-being. However, these differences between correlation coefficients were not formally tested and should not be interpreted as statistically significant differences in association strength. The three well-being dimensions were also positively interrelated. The correlation between social and psychological well-being was particularly strong (r = 0.77), corresponding to approximately 59% shared variance. Because these dimensions were examined as separate outcome variables and were not entered simultaneously as predictors, their association did not create a multicollinearity problem in the mediation models. Nevertheless, the magnitude of the correlation indicates substantial overlap between the two MHC-SF dimensions.

### 3.2. Indirect-Association Analyses

**Figure 1:**
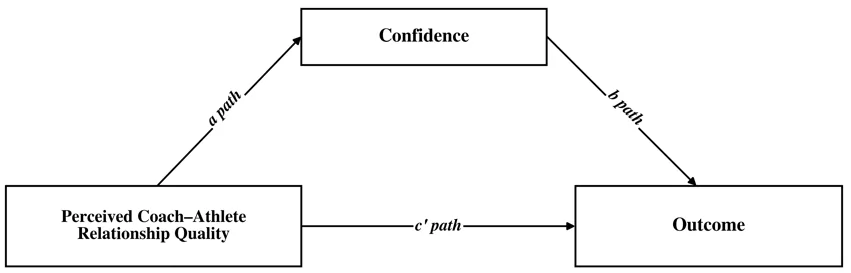
Conceptual statistical model of the indirect associations between perceived coach–athlete relationship quality and emotional, psychological, and social well-being through competitive state self-confidence. Standardized path coefficients (β) for emotional, psychological, and social well-being, respectively, were: *a* = 0.41 in all three models; *b* = 0.26, 0.30, and 0.19; *c* = 0.21, 0.19, and 0.15; *c′* = 0.11, 0.07, and 0.07; and *ab* = 0.11, 0.12, and 0.08. Complete estimates and 95% confidence intervals are reported in Table 3.

Three separate single-mediator models were estimated to examine whether indirect associations between perceived coach–athlete relationship quality and emotional, psychological, and social well-being were observed through competitive state self-confidence. The model estimates are presented in Table 3, and the conceptual statistical model is shown in Figure 1. Perceived coach–athlete relationship quality was positively associated with competitive state self-confidence (B = 0.06, 95% CI [0.04, 0.08], β = 0.41). Competitive state self-confidence was positively associated with emotional well-being (B = 0.44, 95% CI [0.20, 0.68], β = 0.26), psychological well-being (B = 0.47, 95% CI [0.25, 0.69], β = 0.30), and social well-being (B = 0.29, 95% CI [0.07, 0.52], β = 0.19). Positive indirect association estimates through competitive state self-confidence were observed for emotional well-being (B = 0.03, 95% CI [0.01, 0.05], β = 0.11), psychological well-being (B = 0.03, 95% CI [0.01, 0.05], β = 0.12), and social well-being (B = 0.02, 95% CI [0.00, 0.03], β = 0.08). For clarity, the lower bound of the 95% confidence interval for the indirect association with social well-being was 0.008 before rounding and therefore remained above zero. The corresponding direct associations were imprecisely estimated, and their 95% confidence intervals included zero. This pattern was statistically consistent with indirect associations through competitive state self-confidence. However, because the data were cross-sectional, these findings do not establish temporal ordering or demonstrate that competitive state self-confidence constitutes a causal mediating mechanism.

## 4. Discussion

The present study contributes to the growing literature on the interpersonal correlates of athlete mental health by examining the cross-sectional associations among perceived coach–athlete relationship quality, competitive state self-confidence, and emotional, psychological, and social well-being. The findings supported H1 and H2: perceived coach–athlete relationship quality was positively associated with competitive state self-confidence, which was, in turn, positively associated with all three dimensions of well-being. Positive indirect associations through competitive state self-confidence were also observed, consistent with H3. This was statistically consistent with the hypothesized indirect-association model; however, it does not demonstrate a temporal or causal psychological mechanism. Because all variables were assessed at the same time, their temporal ordering and the direction of the observed associations cannot be determined.

The observed associations can be considered alongside prior research on the coach–athlete relationship. A comprehensive review of 25 years of research concluded that closeness, commitment, complementarity, and co-orientation constitute central relational processes through which coaches and athletes influence one another’s development, functioning, and well-being [18]. Contemporary evidence similarly indicates that supportive coaching behaviors and high-quality coach–athlete relationships are associated with greater psychological safety, partly because they create an interpersonal climate in which athletes feel respected, able to express concerns, and less threatened by interpersonal consequences following mistakes or difficulties [11]. Qualitative evidence from former collegiate athletes further suggests that coaches can exert a substantial and enduring influence on athletes’ emotional functioning, perceived support, help-seeking, and broader mental-health experiences [19]. These studies provide theoretical context for the present associations, but the relational processes they describe were not measured directly in this study.

The proposed role of competitive state self-confidence can also be considered in relation to the findings of [9]. In their study, the coach–athlete relationship was associated with self-confidence through perceived coach support and with psychological well-being through received coach support. This prior work offers one possible interpretation of the current indirect associations. However, perceived and received coach support were not measured in the present study, so these processes cannot be established from the current data.

One possible explanation, derived from previous research rather than tested directly here, is that supportive coach–athlete relationships may be associated with competitive state self-confidence by shaping how athletes interpret feedback, mistakes, and performance expectations. Recent evidence indicates that positive coach–athlete relationships are associated with athletes’ self-efficacy, emotional regulation, engagement, and internalization of training goals [20]. Research on supportive coach behavior and psychological safety similarly shows that athletes’ interpretation of coaching behavior depends partly on the quality of the underlying relationship [21]. These findings make this interpretation plausible, but the relevant interpersonal processes were not assessed in the present study.

From a theoretical perspective, the observed associations may also be considered in relation to self-determination theory. Supportive interpersonal environments promote adaptive functioning when they facilitate the satisfaction of the basic psychological needs for autonomy, competence, and relatedness. Recent meta-analytic evidence indicates that autonomy-supportive interpersonal behavior in sport is positively associated with need satisfaction, autonomous motivation, engagement, performance, and well-being [22]. Similarly, autonomy support from coaches and parents has been connected to athlete well-being and performance through adaptive motivational processes [23]. Recent work has also found that psychological-need satisfaction can statistically mediate associations between the coach–athlete relationship and adaptive athlete outcomes [24]. Within this framework, competitive state self-confidence may be viewed as a situation-specific expression of perceived competence. This interpretation remains tentative because psychological-need satisfaction was not measured and temporal ordering cannot be established.

Previous research has identified resilience and dispositional optimism [25], emotional intelligence and athletic engagement [20], and life skills [26] as additional psychological resources associated with athlete outcomes. These variables were not included in the present mediation models and are therefore noted only as direction for future research rather than as explanations tested by the current data.

This broader interpretation is consistent with the Gold Medal Profile for Sport Psychology. The GMP-SP situates the coach–athlete relationship and communication among interpersonal competencies, confidence among fundamental competencies, and mental health as an overarching component of athlete functioning [4]. The present cross-sectional findings can be situated within this framework, but they do not directly test or establish the proposed interpersonal-to-fundamental competency pathway.

Although the MHC-SF theoretically distinguishes social and psychological well-being, these dimensions were strongly correlated in the present sample (r = 0.77), corresponding to approximately 59% shared variance. This substantial overlap should be considered when interpreting dimension-specific results. Future research could examine the discriminant validity of the MHC-SF dimensions in athlete samples using latent-variable methods.

The observed correlation and path coefficients involving psychological well-being were numerically larger than the corresponding coefficients for social well-being; however, these differences were not formally tested. Accordingly, this numerical pattern should not be interpreted as evidence that competitive state self-confidence was significantly more strongly associated with psychological than with social well-being. One possible literature-based interpretation of this numerical pattern is that psychological well-being may be more closely related to competence-relevant functioning, whereas social well-being may depend more strongly on resources extending beyond the coaching dyad; this interpretation was not tested directly in the present study.

This possible interpretation is consistent with a recent systematic review and meta-analysis, which found that social support is positively associated with athlete well-being and negatively associated with anxiety, stress, and depression, while also showing that different sources of support may have distinct associations with mental-health outcomes [3]. Evidence among athletes has similarly linked perceived social support with mental health through additional psychological processes, including resilience and coping resources [27,28].

With appropriate caution, the observed associations suggest that relationship-building and confidence-supportive practices may represent potential targets for coach education. Potential coach-education targets include establishing relationships characterized by trust, mutual respect, and open communication; providing clear and individualized feedback; recognizing effective preparation and improvement; and responding constructively to mistakes. Previous research has associated these practices with a psychologically safer sporting environment in which athletes may feel able to seek support and discuss difficulties [19,21].

However, the present observational findings do not demonstrate that these practices cause improvements in confidence or well-being.

Structured programs such as reROOT demonstrate that autonomy-supportive and relationship-oriented coaching behaviors can be developed through targeted education [29]. Nevertheless, coaches should not be expected to replace qualified mental-health professionals. Confidence-supportive coaching should instead form part of a broader athlete-support system that includes psychological skills development, mental-health awareness, and access to appropriate professional care.

Several limitations should be considered. First, the cross-sectional design does not permit causal or temporal conclusions. The significant indirect associations therefore do not demonstrate that perceived coach–athlete relationship quality causes changes in competitive state self-confidence or well-being. Reverse or reciprocal associations remain possible, and longitudinal or intervention-based studies are needed to establish the direction of these relationships. In addition, the CSAI-2 self-confidence items were completed in a non-standardized competitive context. Participants were not instructed to refer to a specific upcoming competition, and the temporal proximity of questionnaire completion to a competition was not recorded. Competitive state self-confidence referred to an upcoming competitive situation, whereas the MHC-SF assessed well-being during the previous month; this temporal mismatch limits the conceptual alignment of the measures. Second, all variables were assessed using athlete self-report measures, which may have introduced common method variance and shared response tendencies. Future research should incorporate coach reports, behavioral observations, and dyadic assessments. Third, the non-probability convenience-sampling approach and online recruitment may have introduced selection bias. Fourth, the mediation models were not adjusted for potential demographic or sport-related confounding variables. Consequently, the observed associations may partly reflect uncontrolled factors, and the estimates should be interpreted as unadjusted associations.

Athletes who voluntarily completed a survey addressing mental health and related constructs may differ systematically from those who did not participate, which may limit the representativeness and generalizability of the findings.

Because the anonymous survey was not configured to limit participation to one submission per person, potential duplicate submissions could not be verified using personal identifiers, and no formal duplicate-response screening was conducted. An a priori sample-size calculation was also not performed. Competitive state self-confidence was examined as the only intervening variable; other processes—including perceived coach support, psychological need satisfaction, psychological safety, and resilience—may also help explain the association between perceived coach–athlete relationship quality and well-being. Finally, perceived coach–athlete relationship quality was represented by a composite CART-Q score, preventing examination of the distinct roles of closeness, commitment, and complementarity. The heterogeneous sample of Hungarian athletes may also limit generalizability to particular sports, competitive levels, or cultural contexts. Future studies should investigate the CART-Q dimensions separately and examine potential differences according to sport type, competitive level, gender, age, and career stage.

## 5. Conclusions

The present study contributes to a more process-oriented understanding of how the coach–athlete relationship may be associated with athletes’ positive mental health. The analyses showed statistically significant indirect associations between perceived coach–athlete relationship quality and emotional, psychological, and social well-being through competitive state self-confidence. These findings underline the value of developing coaching environments that combine high-quality interpersonal relationships with confidence-supportive practices, while further longitudinal and intervention-based research is needed to clarify the direction and development of these associations over time. (1) More positive athlete perceptions of coach–athlete relationship quality were associated with greater competitive state self-confidence, indicating that the perceived coach–athlete relationship may constitute an important interpersonal context for athletes’ competition-specific confidence. (2) Significant indirect associations through competitive state self-confidence were observed between perceived coach–athlete relationship quality and emotional, psychological, and social well-being. This cross-sectional statistical pattern does not establish temporal or causal mediation. (3) From an applied perspective, the findings emphasize the value of integrating relationship-building and confidence-supportive practices into coach education. However, longitudinal and intervention-based research is needed before causal or temporal conclusions can be established.

## Figures and Tables

**Table 1:** Demographic and Sport-Related Characteristics of the Participants.

No.	Characteristic	Category	N	%	M	SD
1	Age, years	Total sample	211	—	23.39	3.58
2	Sex	Women	149	70.6	—	—
		Men	62	29.4	—	—
3	Sport type	Team sport	101	47.9	—	—
		Individual sport	110	52.1	—	—
		Missing	0	0	—	—
4	Age by sport type, years	Team sport	101	47.9	23.01	3.25
		Individual sport	110	52.1	23.78	3.82
5	Competitive level	Semi-professional	92	43.6	—	—
		Amateur	84	39.8	—	—
		Professional	35	16.6	—	—
		Missing	0	0	—	—
6	Weekly training volume, hours	Total sample	211	—	11.75	8.94

Note. N = 211. M = mean; SD = standard deviation. Percentages were calculated using the total sample and may not total 100 due to rounding. Sport type was missing for one participant. Years of sport experience were not assessed.

**Table 2:** Means, standard deviations, internal consistencies, and correlations among the study variables.

No.	Variable	N	M	SD	α	1	2	3	4	5
1	Perceived coach–athlete relationship quality	211	13.32	4.32	0.97	—				
2	Competitive state self-confidence	211	2.71	0.67	0.85	0.41 ***	—			
3	Emotional well-being	211	4.42	1.15	0.87	0.21 **	0.30 ***	—		
4	Social well-being	211	4.32	1.04	0.75	0.15 *	0.22 **	0.60 ***	—	
5	Psychological well-being	211	4.59	1.05	0.83	0.19 **	0.33 ***	0.63 ***	0.77 ***	—

Note. N = 211. The 95% confidence intervals for the means were [12.73, 13.91] for perceived coach–athlete relationship quality, [2.62, 2.80] for competitive state self-confidence, [4.26, 4.58] for emotional well-being, [4.18, 4.46] for social well-being, and [4.45, 4.73] for psychological well-being. The theoretical score ranges were 3–21 for perceived coach–athlete relationship quality, 1–4 for competitive state self-confidence, and 1–6 for each MHC-SF well-being dimension. The perceived coach–athlete relationship quality composite score was calculated by summing the mean scores for closeness, commitment, and complementarity. * *p* < 0.05, ** *p* < 0.01, *** *p* < 0.001.

**Table 3:** Indirect Associations Between Perceived Coach–Athlete Relationship Quality and Mental Health Through Competitive State Self-Confidence.

Outcome	Effect	B	SE	95% CI	β	*z*	*p*
Emotional well-being	a path	0.06	0.01	[0.04, 0.08]	0.41	6.55	<0.001
	b path	0.44	0.12	[0.20, 0.68]	0.26	3.57	<0.001
	Indirect effect	0.03	0.01	[0.01, 0.05]	0.11	3.13	0.002
	Direct effect	0.03	0.02	[−0.01, 0.07]	0.11	1.52	0.129
	Total effect	0.06	0.02	[0.02, 0.09]	0.21	3.17	0.002
Psychological well-being	a path	0.06	0.01	[0.04, 0.08]	0.41	6.55	<0.001
	b path	0.47	0.11	[0.25, 0.69]	0.30	4.21	<0.001
	Indirect effect	0.03	0.01	[0.01, 0.05]	0.12	3.54	<0.001
	Direct effect	0.02	0.02	[−0.02, 0.05]	0.07	1.00	0.318
	Total effect	0.05	0.02	[0.01, 0.08]	0.19	2.87	0.004
Social well-being	a path	0.06	0.01	[0.04, 0.08]	0.41	6.55	<0.001
	b path	0.29	0.11	[0.07, 0.52]	0.19	2.58	0.010
	Indirect effect	0.02	0.01	[0.00, 0.03]	0.08	2.40	0.016
	Direct effect	0.02	0.02	[−0.02, 0.05]	0.07	1.01	0.313
	Total effect	0.04	0.02	[0.00, 0.07]	0.15	2.23	0.026

Note. Path a represents the association between perceived coach–athlete relationship quality and competitive state self-confidence. Path b represents the association between competitive state self-confidence and the relevant well-being outcome while controlling for perceived coach–athlete relationship quality. The direct effect represents the association between perceived coach–athlete relationship quality and the relevant well-being outcome while controlling for competitive state self-confidence. The total effect represents the association without controlling for competitive state self-confidence. The indirect effect is represented by the product of paths a and b. B = unstandardized effect estimate; SE = standard error; β = standardized effect estimate; CI = confidence interval.

## Data Availability

The anonymized dataset and Jamovi analysis files are not publicly available because public data deposition was not explicitly covered by the ethical approval and participant consent. These materials may be obtained from the corresponding author upon reasonable request, subject to applicable institutional, ethical, and data-protection requirements. The variable-scoring procedures are described in the Section 2.3.

## References

[1] Kuettel A., Larsen C.H. (2020). Risk and Protective Factors for Mental Health in Elite Athletes: A Scoping Review. Int. Rev. Sport Exerc. Psychol..

[2] Stevens M., Cruwys T., Olive L., Rice S. (2024). Understanding and Improving Athlete Mental Health: A Social Identity Approach. Sports Med..

[3] Luo J., Du R., Wang X., Luo L. (2025). The Relationship between Social Support and Mental Health in Athletes: A Systematic Review and Meta-Analysis. Front. Psychol..

[4] Durand-Bush N., Baker J., Berg F., Richard V., Bloom G. (2023). The Gold Medal Profile for Sport Psychology (GMP-SP). J. Appl. Sport Psychol..

[5] Martens R., Burton D., Vealey R.S., Bump L.A., Smith D.E., Martens R., Vealey R.S., Burton D. (1990). Development and Validation of the Competitive State Anxiety Inventory-2 (CSAI-2). Competitive Anxiety in Sport.

[6] Tóth R., Sipos K., Tóth L. (2025). A Magyar Versenyszorongás Skála (CSAI-2HR) validitás- és reliabilitásvizsgálata, valamint standard értékeinek meghatározása. Magy. Pszichol. Szle..

[7] Vealey R.S., Garner-Holman M., Hayashi S.W., Giacobbi P. (1998). Sources of Sport-Confidence: Conceptualization and Instrument Development. J. Sport Exerc. Psychol..

[8] Jekauc D., Fiedler J., Wunsch K., Mülberger L., Burkart D., Kilgus A., Fritsch J. (2025). The Effect of Self-Confidence on Performance in Sports: A Meta-Analysis and Narrative Review. Int. Rev. Sport Exerc. Psychol..

[9] Coussens A.H., Stone M.J., Donachie T.C. (2025). Coach–Athlete Relationships, Self-Confidence, and Psychological Wellbeing: The Role of Perceived and Received Coach Support. Eur. J. Sport Sci..

[10] Jowett S., Ntoumanis N. (2004). The Coach–Athlete Relationship Questionnaire (CART-Q): Development and Initial Validation. Scand. J. Med. Sci. Sports.

[11] Şenel E., Küttel A., Adiloğulları İ., Jowett S. (2025). Psychological Predictors of Mental Well-Being in Judo Athletes: Exploring the Impacts of the Coach–Athlete Relationship, Social Support, and Psychological Safety. Psychol. Sport Exerc..

[12] Keyes C.L.M. (2002). The Mental Health Continuum: From Languishing to Flourishing in Life. J. Health Soc. Behav..

[13] Krisztina K., Rita F.F., Noémi G. (2021). Az Edző–Sportoló Kapcsolat Kérdőív hazai adaptációja, a sportoló szemszögén keresztül. Magy. Sport. Szle..

[14] Yang S.X., Jowett S. (2013). The Psychometric Properties of the Short and Long Versions of the Coach–Athlete Relationship Questionnaire. Meas. Phys. Educ. Exerc. Sci..

[15] Lafrenière M.-A.K., Jowett S., Vallerand R.J., Carbonneau N. (2011). Passion for Coaching and the Quality of the Coach–Athlete Relationship: The Mediating Role of Coaching Behaviors. Psychol. Sport Exerc..

[16] Keyes C.L.M., Wissing M., Potgieter J.P., Temane M., Kruger A., Van Rooy S. (2008). Evaluation of the Mental Health Continuum–Short Form (MHC-SF) in Setswana-Speaking South Africans. Clin. Psychol. Psychother..

[17] Reinhardt M., Horváth Z., Tóth L., Kökönyei G. (2020). A Mentális Egészség Kontinuum Skála rövid változatának hazai validációja. Magy. Pszichol. Szle..

[18] Jowett S. (2025). 25 Years of Relationship Research in Sport: The Quality of the Coach–Athlete Relationship as Defined by Closeness, Commitment, Complementarity and Co-Orientation (3+1Cs Model). Psychol. Sport Exerc..

[19] Kernan A.R., Cope M.R., Jarvis J.A., Dufur M.J. (2025). Coach–Athlete Relationships and Mental Health: An Exploratory Study on Former Female NCAA Student-Athletes. Int. J. Environ. Res. Public Health.

[20] Pan F., Sui W. (2025). Research on Coach–Athlete Relationship and Team Performance Based on 3Cs Theory: The Chain Mediating Role of Emotional Intelligence and Athletic Engagement. Front. Psychol..

[21] Şenel E., Jowett S., Adiloğulları İ., Kerr-Cumbo R. (2025). Investigating the Impact of Coach Behaviours and Coach–Athlete Relationships on Psychological Safety. Int. J. Sport Exerc. Psychol..

[22] Mossman L.H., Slemp G.R., Lewis K.J., Colla R.H., O’Halloran P. (2024). Autonomy Support in Sport and Exercise Settings: A Systematic Review and Meta-Analysis. Int. Rev. Sport Exerc. Psychol..

[23] Lemelin E., Verner-Filion J., Carpentier J., Carbonneau N., Mageau G.A. (2022). Autonomy Support in Sport Contexts: The Role of Parents and Coaches in the Promotion of Athlete Well-Being and Performance. Sport Exerc. Perform. Psychol..

[24] Zhang R., Rhim Y.-T. (2024). The Effect of Coach–Athlete Relationships on Motor Behaviour in College Athletes—Mediating Effects of Psychological Needs. Behav. Sci..

[25] Zhang N., Du G., Tao T. (2025). Empowering Young Athletes: The Influence of Autonomy-Supportive Coaching on Resilience, Optimism, and Development. Front. Psychol..

[26] Tozetto W.R., Lima C.O.V., De Sousa Ferreira Dos Santos F., Durand-Bush N., Milistetd M. (2026). The Mediating Role of Life Skills in the Association of the Coach–Athlete Relationship with Youth Athletes’ Well-Being. Discov. Ment. Health.

[27] Liu Z., Zhao X., Zhao L., Zhang L. (2023). Relationship between Perceived Social Support and Mental Health among Chinese College Football Athletes: A Moderated Mediation Model. BMC Psychol..

[28] Mira T., Jacinto M., Costa A.M., Monteiro D., Diz S., Matos R., Antunes R. (2023). Exploring the Relationship between Social Support, Resilience, and Subjective Well-Being in Athletes of Adapted Sport. Front. Psychol..

[29] Lemelin E., Carpentier J., Gadoury S., Petit É., Forest J., Richard J.-P., Joussemet M., Mageau G.A. (2024). The reROOT Coaching Program: A Pilot Randomized Controlled Trial Evaluating Its Impact on Coaching Style and Athlete Sports Development. Int. Sport Coach. J..

